# Assessment of *Mycobacterium tuberculosis* transmission in Oxfordshire, UK, 2007–12, with whole pathogen genome sequences: an observational study

**DOI:** 10.1016/S2213-2600(14)70027-X

**Published:** 2014-03-04

**Authors:** Timothy M Walker, Maeve K Lalor, Agnieszka Broda, Luisa Saldana Ortega, Marcus Morgan, Lynne Parker, Sheila Churchill, Karen Bennett, Tanya Golubchik, Adam P Giess, Carlos Del Ojo Elias, Katie J Jeffery, Ian C J W Bowler, Ian F Laurenson, Anne Barrett, Francis Drobniewski, Noel D McCarthy, Laura F Anderson, Ibrahim Abubakar, H Lucy Thomas, Philip Monk, E Grace Smith, A Sarah Walker, Derrick W Crook, Tim E A Peto, Christopher P Conlon

**Affiliations:** **Nuffield Department of Medicine, University of Oxford, Oxford, UK**(T M Walker MRCP, T Golubchik PhD, A P Giess MSc, C Del Ojo Elias MSc, A S Walker PhD, Prof D W Crook FRCPath, Prof T E A Peto FRCP, C P Conlon FRCP); **Department of Microbiology and Infectious Disease, Oxford University Hospitals NHS Trust, Oxford, UK** (T M Walker, M Morgan MSc, K J Jeffery FRCPath, I C J W Bowler FRCPath, Prof D W Crook, Prof T E A Peto, C P Conlon); **Public Health England TB Section, Centre for Infectious Disease Surveillance and Control, Colindale, London, UK** (M K Lalor PhD, L F Anderson PhD, Prof I Abubakar FRCP, H L Thomas MFPH); **Public Health England National Mycobacterial Reference Laboratory, Queen Mary’s School of Medicine and Dentistry, London, UK** (A Broda MRes**,** Prof F Drobniewski FRCPath); **Thames Valley Public Health England Centre, Chilton, UK** (L Saldana Ortega MSc, N D McCarthy (DPhil); **Oxford Health NHS Foundation Trust, Oxford, UK** (L Parker BA, S Churchill BSc, K Bennett BA); **Scottish Mycobacteria Reference Laboratory, Royal Infirmary of Edinburgh, Edinburgh, UK** (I F Laurenson FRCPath); **Public Health England Newcastle Laboratory, Freeman Hospital, Newcastle upon Tyne, UK** (A Barrett BSc); **Public Health England, East Midlands Centre, Nottingham, UK** (P Monk FFPHM); **Public Health England West Midlands Public Health Laboratory, Birmingham, UK** (E G Smith FRCPath); **Oxford National Institute for Health Research Biomedical Research Centre, John Radcliffe Hospital, Oxford, UK** (A S Walker, Prof D W Crook, Prof T E A Peto); **Centre for Infectious Disease Epidemiology and MRC Clinical Trials Unit, University College London, London, UK** (Prof I Abubakar); **and Department of Infectious Diseases, Imperial College, London, UK** (Prof F Drobniewski, A Broda)

## Abstract

**Background:**

Patients born outside the UK have contributed to a 20% rise in the UK’s tuberculosis incidence since 2000, but their effect on domestic transmission is not known. Here we use whole-genome sequencing to investigate the epidemiology of tuberculosis transmission in an unselected population over 6 years.

**Methods:**

We identified all residents with Oxfordshire postcodes with a *Mycobacterium tuberculosis* culture or a clinical diagnosis of tuberculosis between Jan 1, 2007, and Dec 31, 2012, using local databases and checking against the national Enhanced Tuberculosis Surveillance database. We used Illumina technology to sequence all available *M tuberculosis* cultures from identified cases. Sequences were clustered by genetic relatedness and compared retrospectively with contact investigations. The first patient diagnosed in each cluster was defined as the index case, with links to subsequent cases assigned first by use of any epidemiological linkage, then by genetic distance, and then by timing of diagnosis.

**Findings:**

Although we identified 384 patients with a diagnosis of tuberculosis, country of birth was known for 380 and we sequenced isolates from 247 of 269 cases with culture-confirmed disease. 39 cases were genomically linked within 13 clusters, implying 26 local transmission events. Only 11 of 26 possible transmissions had been previously identified through contact tracing. Of seven genomically confirmed household clusters, five contained additional genomic links to epidemiologically unidentified non-household members. 255 (67%) patients were born in a country with high tuberculosis incidence, conferring a local incidence of 109 cases per 100 000 population per year in Oxfordshire, compared with 3·5 cases per 100 000 per year for those born in low-incidence countries. However, patients born in the low-incidence countries, predominantly UK, were more likely to have pulmonary disease (adjusted odds ratio 1·8 [95% CI 1·2–2·9]; p=0·009), social risk factors (4·4 [2·0–9·4]; p<0·0001), and be part of a local transmission cluster (4·8 [1·6–14·8]; p=0·006).

**Interpretation:**

Although inward migration has contributed to the overall tuberculosis incidence, our findings suggest that most patients born in high-incidence countries reactivate latent infection acquired abroad and are not involved in local onward transmission. Systematic screening of new entrants could further improve tuberculosis control, but it is important that health care remains accessible to all individuals, especially high-risk groups, if tuberculosis control is not to be jeopardised.

## Introduction

The burden of tuberculosis in the UK is among the highest in western Europe, with about 9000 new cases per year.^1^ The incidence has risen by more than 20% since 2000, from 11·4 to 13·9 cases per 100 000 population.^2^ Although the additional cases are accounted for by patients who were born overseas,^3^ the role of these patients in local transmission remains unclear. Designing control measures to reverse the increase in incidence requires an improved understanding of the contributions of reactivated versus newly acquired infections to overall disease.

The UK National Strain Typing Project introduced routine 24-locus mycobacterial interspersed repetitive unit-variable number tandem repeats (MIRU-VNTR) genotyping in 2010,^4^ but this method cannot be used to reliably distinguish recent from past transmission.^5^ The results of several studies have shown the additional resolution provided by use of whole-genome sequencing (WGS) of *Mycobacterium tuberculosis* in outbreak settings,^5-7^ but this technique has yet to be applied to study an unselected, geographically restricted population. Oxfordshire (population 760 000) is a low-incidence region (8·4 cases per 100 000 population) where, like the UK as a whole, most cases are restricted to a small number of urban areas.^2,8^ Here we use WGS to investigate the incidence of tuberculosis arising from transmission in Oxfordshire and whether transmission varies between people born in high-incidence versus low-incidence settings.

## Methods

### Case identification and sample selection

We identified all residents with Oxfordshire postcodes with an *M tuberculosis* culture or a clinical diagnosis of tuberculosis between Jan 1, 2007, and Dec 31, 2012, from three sources. Relevant diagnostic codes and microbiology results were identified from the Oxford University Hospitals (OUH) Patient Safety Server, a warehouse of all microbiology tests and OUH admissions. The OUH Trust provides all microbiology laboratory services, more than 99% of acute care, and more than 90% of specialist services in the county. Additionally, we reviewed all records kept by the Thames Valley Health Protection Unit, Chilton, UK, and local specialist tuberculosis nurses. All identified cases were then checked against the national Enhanced Tuberculosis Surveillance (ETS) database. Additional demographic (age, sex, social risk factors, year of UK entry, and country of birth), clinical (pulmonary *vs* non-pulmonary), and microbiological data (microscopy and culture results) were also obtained from ETS.

At least one *M tuberculosis* complex culture was sought for each patient with microbiologically confirmed disease. Where possible, cultures were obtained from the OUH microbiology laboratory (from a frozen archive 2007–10 and obtained prospectively from 2011 onwards). Cultures referred to other UK hospitals were retrieved from the mycobacterial reference laboratories in London, Birmingham, Newcastle, or Edinburgh.

### DNA preparation and sequencing

All cultures were grown in Mycobacterial Growth Indicator Tubes (Becton–Dickinson, Oxford, UK) containing modified Middlebrooks 7H9 liquid medium and on Löwenstein–Jensen agar (Media for Mycobacteria, Wales, UK). Mature cultures were suspended in 400 μL of 0·85% saline, sonicated at 35 kHz for 20 min and heated to 95°C for 2 h to render them non-viable. DNA was extracted and purified by use of the Fuji Quickgene kit (Kurabo Bio-Medical, Osaka, Japan) with an added mechanical disruption step using the Fastprep homogeniser and Lysing Matrix B (MP Biomedicals, Santa Ana, CA, USA). All samples were processed in Oxford except where the Public Health England National Mycobacterial Reference Unit, London, processed archived samples with the cetyltrimethylammonium bromide method.^9^

Libraries were prepared by use of Nextera kits and sequenced on HiSeq platforms (both Illumina, San Diego, CA, USA) at the Wellcome Trust Centre for Human Genetics, Oxford. Paired-end reads were mapped with Stampy (version 1.0.22)^10^ to the H37Rv (GenBank NC000962.2) reference genome as previously described.^5^ 7·4% of the H37Rv genome was identified as repetitive by use of self–self BLAST and was masked. Variant calls in non-repetitive regions were made with SAMtools mpileup (version 0.1.18),^11^ providing they were supported by at least five reads, including one in each direction. Sites where minority variants represented more than 10% of read depth were defined as mixed and no base called. Mean high-quality read-depth was 106 (range 25–195). Within-strain read depth varied with a range of SD of 9–55. After update of the previous bioinformatics mapping and filtering processes, sites in the top 2·5 percentiles of read depth and within 12 nucleotides of another variant were included, which increased mean coverage from 88% to 92% of the genome without affecting previously described thresholds of genetic relatedness or introducing false-positive variant calls.^5^ Consistency in variant calling was assessed by resequencing isolates on different flow cells as technical replicates. Pairwise comparisons were used to identify only a single false-positive variant call across 202 genomes. Short-read data were deposited in the European Nucleotide Archive.

### Epidemiological and genomic cluster analysis

The specialist tuberculosis nurses (LP, SC, and KB), lead infectious diseases physician for the Oxfordshire tuberculosis service (CPC), and local consultant for communicable disease control (NDM) independently identified epidemiological linkage, defined as shared space and time, masked to WGS, with discrepancies resolved by consensus. Assessments were according to previous national guideline-directed cluster investigations.^12,13^ Genomic clusters were ascertained independently of the epidemiological data, and were defined where no more than 12 single-nucleotide polymorphisms (SNPs) separated a patient isolate from that of at least one other patient in the cluster. For our sequence assembling and filtering pipeline, 12 SNPs were the previously defined upper threshold of genomic relatedness noted within hosts and between epidemiologically related hosts.^5^ Plausible transmission networks were constructed for each genomic cluster, and epidemiological cluster with available sequence data, as previously described.^5^ Briefly, the first patient diagnosed in each cluster was defined as the index case, with links to subsequent cases assigned first by use of any epidemiological linkage, then by genetic distance, and then by timing of diagnosis. Hence, the total number of links in each cluster is the number of patients in that cluster minus the index case.

Incidence was calculated according to the postcode with denominator data from the Office of National Statistics. Incidence specific to country of birth was calculated using a regional denominator from the Office of National Statistics because data for countries of birth by postcode were unavailable. Countries of birth with an incidence of tuberculosis of more than 50 cases per 100 000 population per year were classified as high incidence, and those below this threshold were classified as low incidence.^14^ Phylogenetic trees were built in PhyML^15^ (version 3.0) using a generalised time reversible model and whole genomes under the assumption that null calls at non-variant positions were the same as the reference. The association between high-incidence versus low-incidence country of birth and disease characteristics or clustering by epidemiology or genomics was assessed with logistic regression in Stata (version 13.1). All analyses were adjusted for age and sex (availability of the other factors varied).

The Health Protection Regulations 2010 require the notification of all tuberculosis cases, and the 2003 Health Protection Agency Act and 2002 Statutory Instrument 1438 provide legislative cover to undertake follow-up of notified cases of tuberculosis, including their contacts. Because this study was done jointly with Public Health England as an assessment of service delivery, including contact tracing, no research ethics committee application was required.^5^

### Role of the funding source

The sponsors of the study had no role in the study design, gathering, analysis, or interpretation of data, or the writing of the report. MKL, LFA, IA, HLT, TEAP, and ASW had access to the demographic data; LSO, LP, SC, KB, NDM, and CPC had access to the epidemiological data; ABr, MM, KJJ, ICJWB, IFL, ABa, FD, EGS, ASW, TEAP, DWC, and CPC had access to components of the microbiological data; TG, APG, and CDOE had access to the WGS data. The corresponding author had full access to all the data and the final responsibility to submit for publication.

## Results

**Figure 1:**
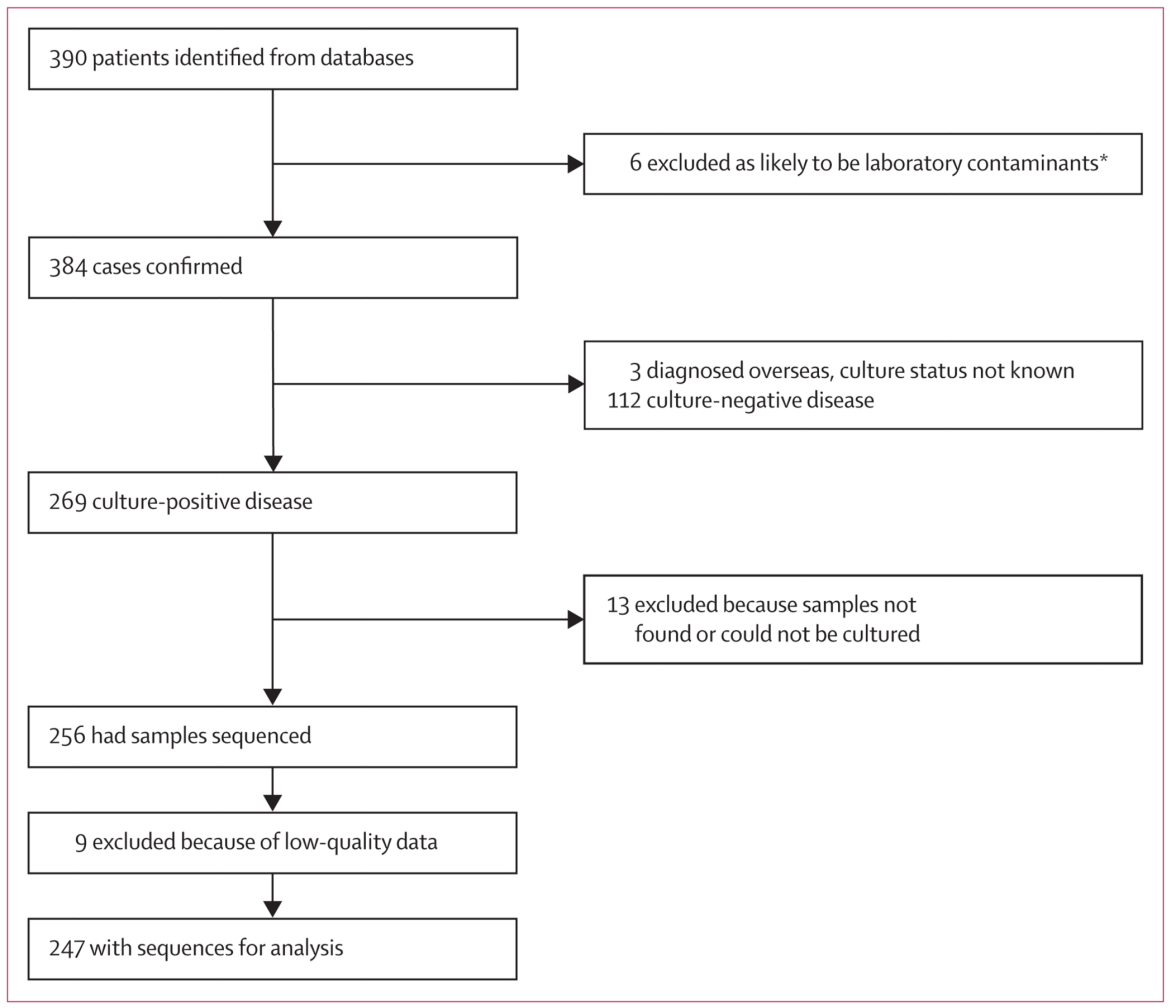
Flow chart of sample selection *Three laboratory contaminants were identified previously and three by use of whole-genome sequencing.

390 Oxfordshire residents had an *M tuberculosis* culture or clinical diagnosis of tuberculosis. Six of these individuals were excluded because the isolates from them were thought to be laboratory contaminants, leaving 384 cases. 269 patients had culture-positive disease and 112 had culture-negative disease, and the status of three patients diagnosed overseas could not be ascertained (figure 1). 22 (8%) of 269 isolates could not be cultured or retrieved, or failed WGS quality control, leaving 247 patients with available sequence data (figure 1).

**Figure 2:**
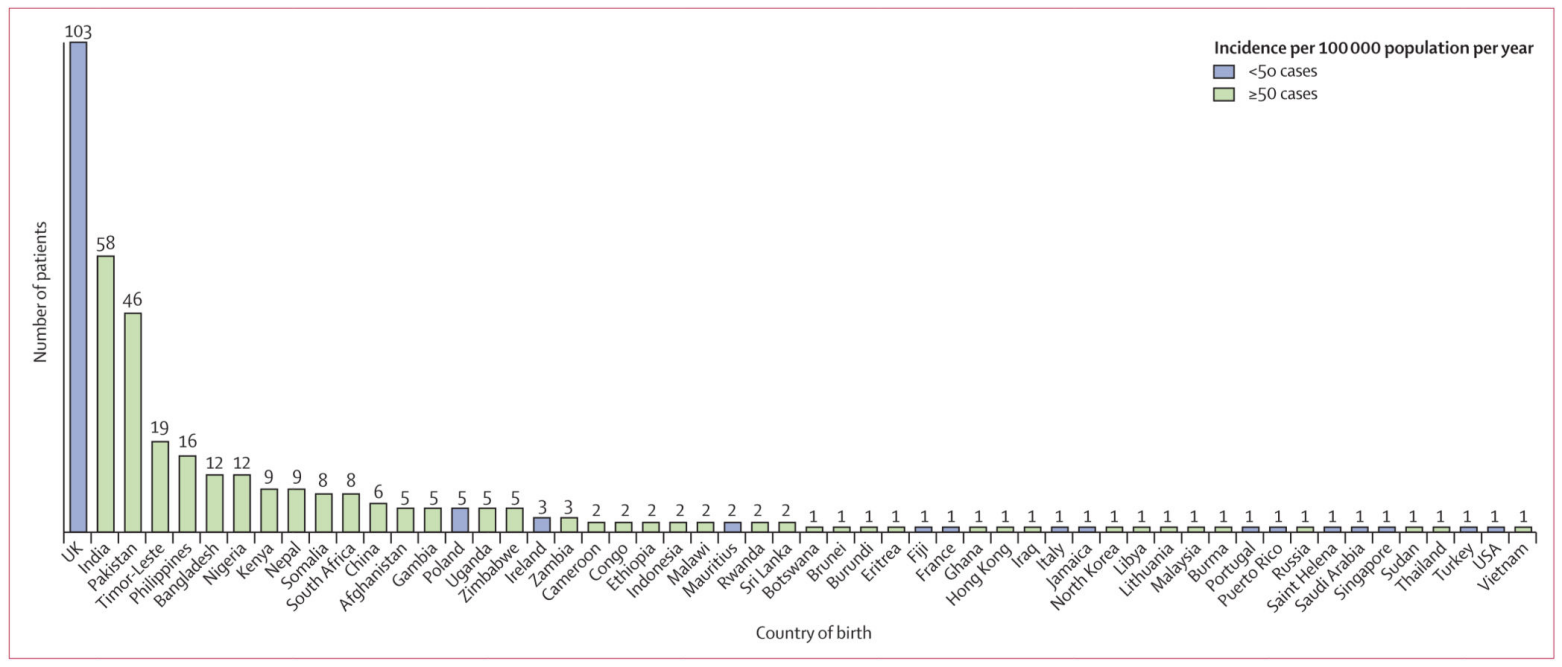
Country of birth of patients with tuberculosis in Oxfordshire, UK, 2007–12 Country of birth was not known for four patients. High and low incidences defined according to WHO. ^14^

**Figure 3:**
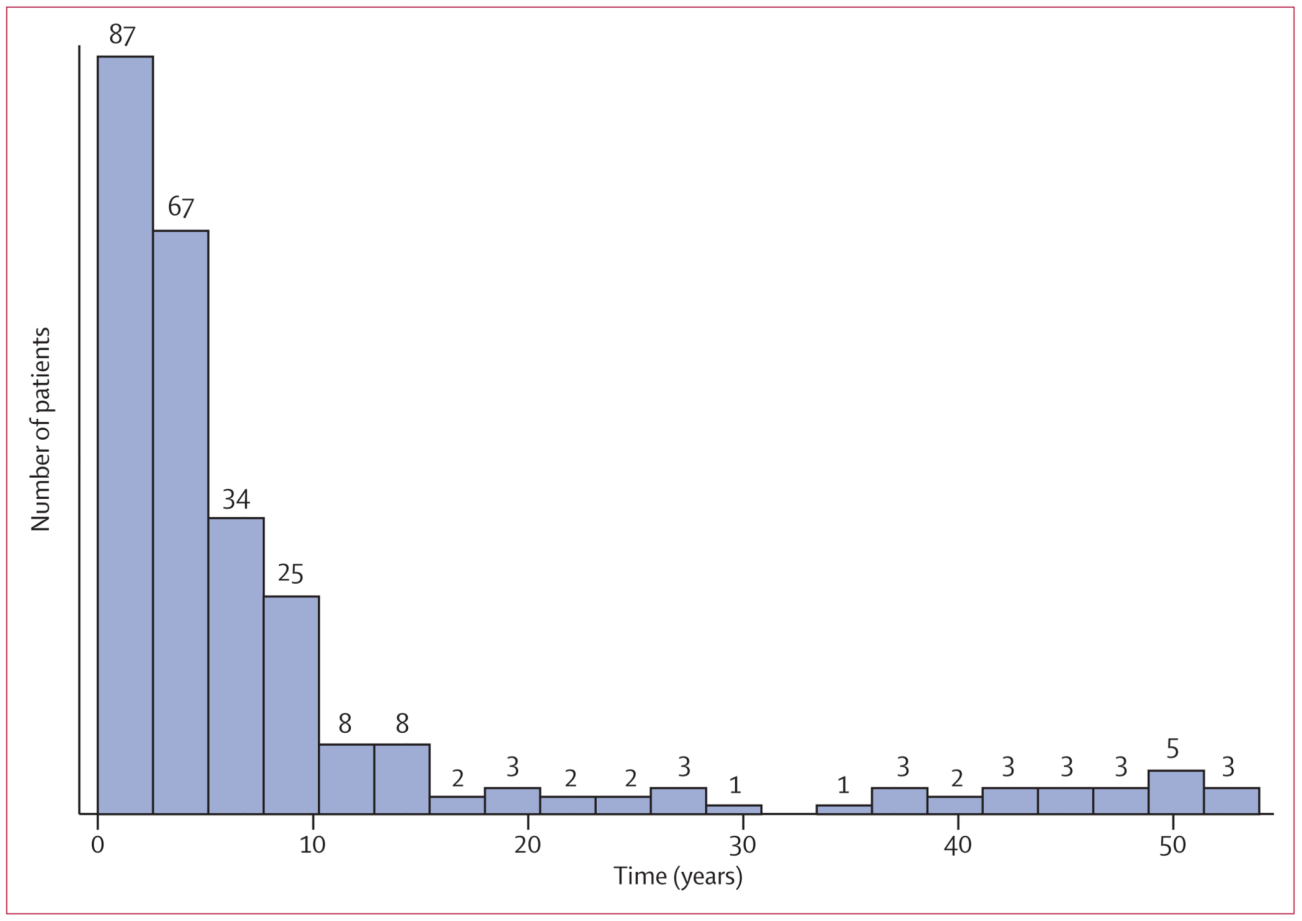
Time interval between entry to the UK and diagnosis of tuberculosis Data for year of entry to the UK were not available for 12 patients.

**Figure 4:**
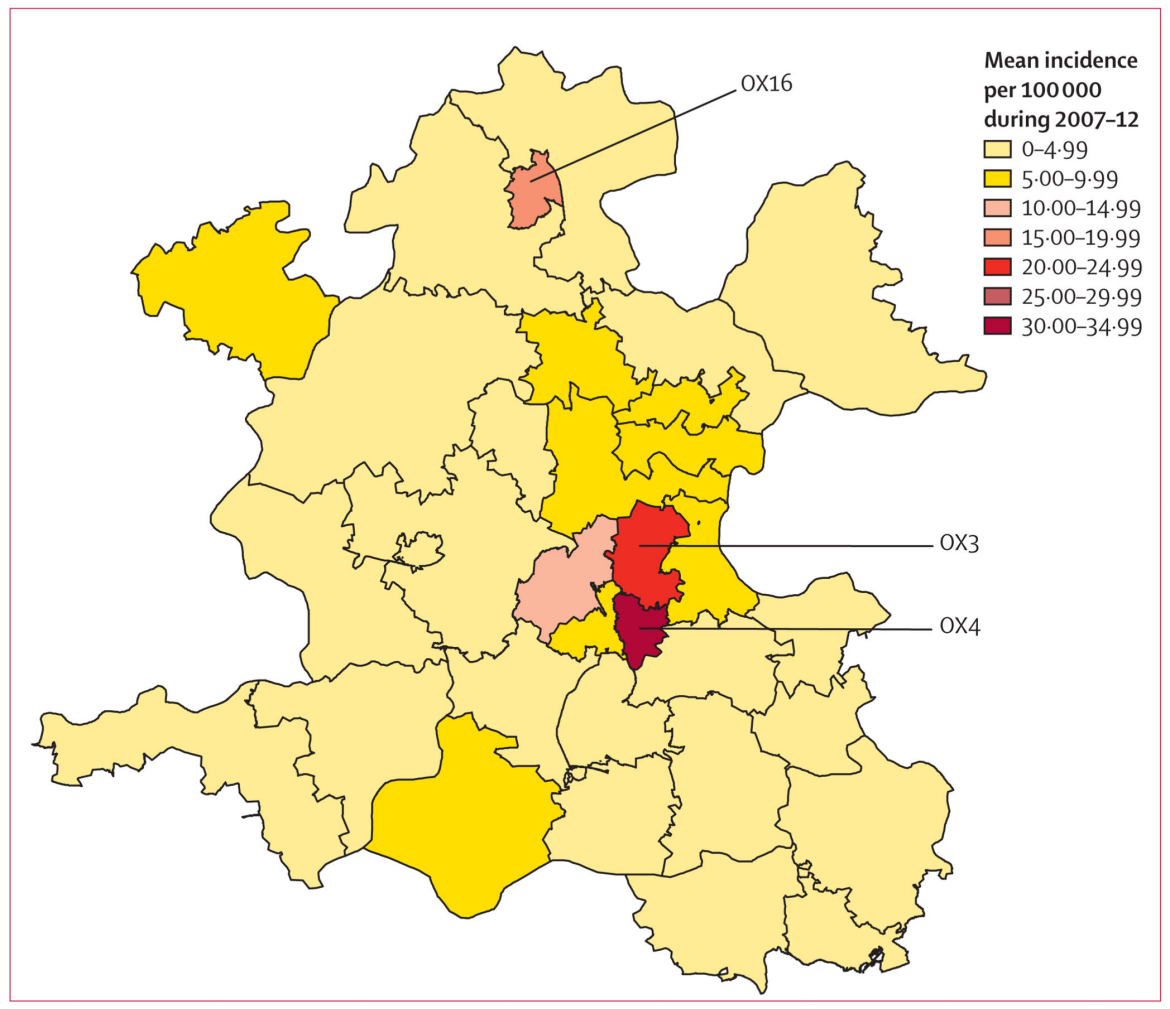
Mean tuberculosis incidence in Oxfordshire (2007–12) Map based on 383 of 384 cases: the postcode for one patient was unknown. Crown copyright and database rights 2013 Ordnance Survey 100016969.

Median age of patients was 34 years (range 1–89), with 255 (67%) of 380 patients born in a high-incidence country, 103 (27%) in the UK, and 22 (6%) in another low-incidence country (figure 2). The place of birth was not known for four patients, including one with culture-positive disease. For non-UK-born patients, a median 5 years (IQR 2–9) had elapsed since entry to the UK to tuberculosis diagnosis (figure 3). The 6% of Oxfordshire’s population born in a high-incidence country had a tuberculosis incidence of 109 cases per 100 000 population per year compared with 3·5 cases per 100 000 per year for those born in a low-incidence country (3·0 cases per 100 000 if UK born and 7·2 per 100 000 if born in another low-incidence country). Three postcodes (OX3 [n=43 000], OX4 [n=62 000], and OX16 [n=47 000]) accounted for 20% of the Oxfordshire population and 233 (61%) of 383 cases for whom the postcode was known (figure 4). 178 (77%) of 232 cases residing in these postcode areas were born in high-incidence countries (country of origin was unknown for one patient) versus 77 (52%) of 148 who were living elsewhere (p<0·0001).

197 (52%) of 380 evaluable patients had pulmonary disease (four cases had unknown site of disease), and those born in a low-incidence country were more likely to have pulmonary disease (odds ratio [OR] 1·8, 95% CI 1·2–2·9; p=0·009), but less likely to have culture-positive disease (0·6, 0·4–0·99; p=0·045; table). Social risk factors (alcohol or drug misuse, homelessness, or time served in prison) were present in 36 (14%) of 261 evaluable patients, and were also more prevalent in those born in low-incidence countries (4·4, 2·0–9·4; p<0·0001; table). There was no difference in the proportion of patients with available data for social risk factors from high-incidence and low-incidence countries of birth (87 [70%] of 125 and 174 [68%] of 255 cases, respectively; p=0·81).

**Figure 5:**
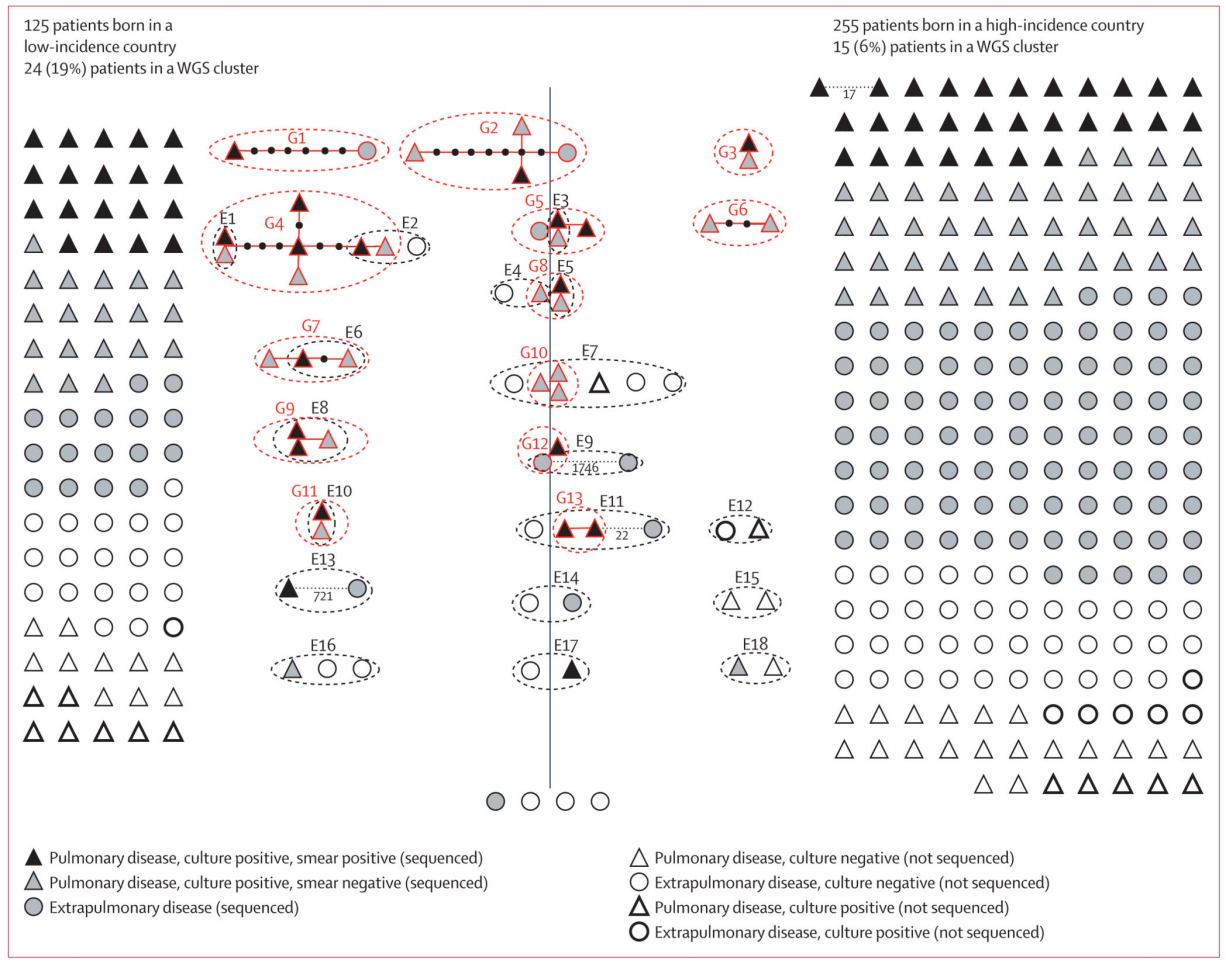
All cases in Oxfordshire, UK, (2007–12) by incidence in country of birth, and by epidemiological and genomic clustering Patients born in low-incidence countries are on the left and those born in high-incidence countries are on the right of the figure. Four patients whose country of birth was not known are at the bottom centre of the figure. Each shape (triangle or circle) represents a patient. Epidemiological clusters (E1–18) are circled in black and genetic links, shown as networks with edges representing the genetic distance, are circled in red. Edges in networks are red for distances within 12 SNPs. Genetic links of interest but greater than 12 SNPs are indicated by black dashed lines, representing the SNP distances. Patients in WGS clusters who are zero SNPs apart are indicated by shapes that abut each other, whereas distances of at least 1 SNP are quantified by the number of red lines (separated by small black nodes if >1 SNP) between patients. Epidemiological or WGS clusters that include patients born in low-incidence countries and patients born in high-incidence countries cross the central vertical line. SNP=single-nucleotide polymorphism. WGS=whole-genome sequencing.

Epidemiological investigations had identified 18 epidemiological clusters (E1–E18) with 46 patients, accounting for 28 potential transmission events within Oxfordshire over 6 years (figure 5). All but two epidemiological links were between family members and the remaining hypothesised transmissions occurred in a school (E8) and in the community (E10; figure 5). MIRU-VNTR was introduced in 2010, but did not result in the identification of any additional epidemiological clusters. Although ten of 18 epidemiologically defined clusters had patients born in high-incidence countries, cases born in low-incidence countries were more likely to be identified as part of an epidemiological cluster (OR 3·3, 95% CI 1·4–7·8; p=0·006), independently of potentially confounding social risk factors (adjusted OR 3·0, 1·2–7·2; p=0·016; table). No significant differences were noted in the odds of pulmonary disease, social risk factors, or epidemiological linkage between UK-born patients and those born in other low-incidence countries (p>0·45; appendix p 3), or in age (rank sum p=0·99; appendix p 4).

Children (aged <18 years) were more likely to be born in a low-incidence country (p=0·001), and, as expected, were more likely to have culture-negative disease (p=0·003), and to be epidemiologically linked to a cluster (household or school; p<0·0001), although six of 13 UK-born patients younger than 10 years were not epidemiologically linked to another case (data not shown).

Assessing pairwise nucleotide differences in the 247 patients with culture-confirmed disease and whole-genome sequences, the isolates from 39 patients were within 12 SNPs of another isolate, forming 13 genomic clusters (G1–G13) with 26 plausible transmission events (figure 5). The remaining 208 (84%) patients could not be genomically linked to another within the 6-year study. Patients born in low-incidence countries were more likely to be genomically linked to another case (OR 5·8, 2·7–12·4; p<0·0001), even after adjustment for social risk factors (4·8, 95% CI 1·6–14·8; p=0·006; table). For patients born in low-incidence countries, estimates suggested that UK-born patients were more likely to be genomically linked to a cluster but numbers were too few to exclude this finding being due to chance (2·0; p=0·45; appendix).

**Figure 6:**
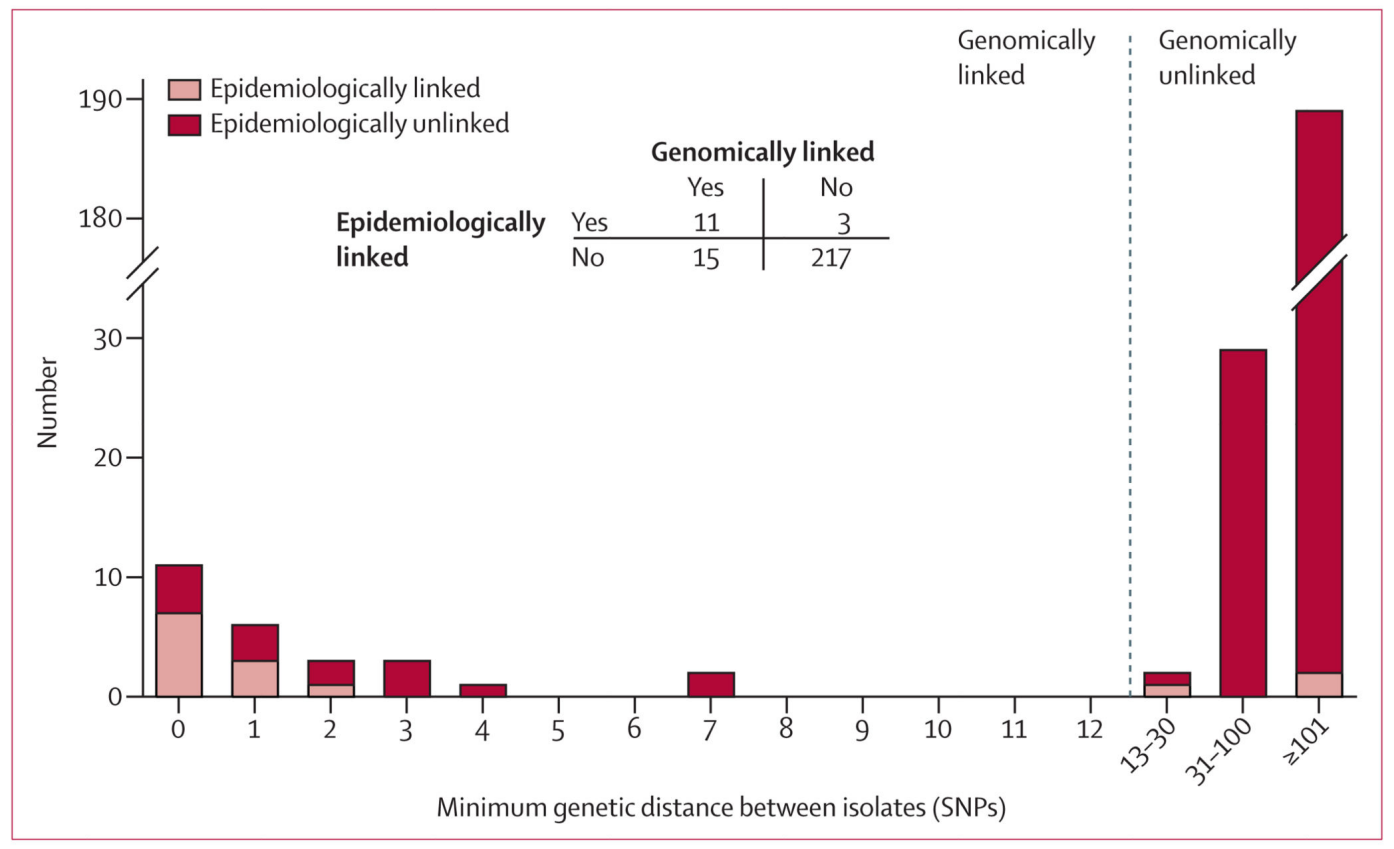
Minimum genetic distance between isolates

**Figure 7:**
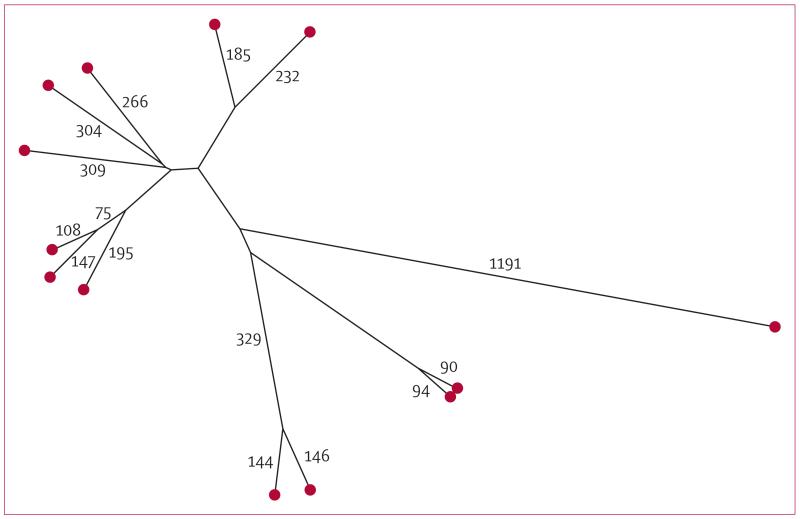
Phylogenetic relations between whole-genome-sequencing clusters Maximum likelihood tree of 13 clusters as ascertained with whole genome sequencing are represented by red circles. SNP distances are annotated on the branches. SNP=single-nucleotide polymorphism.

Actual differences within genomic clusters ranged from zero to seven SNPs (median 1 SNP, IQR 0–2), despite a predefined upper limit of 12 SNPs (figure 6). After exclusion of secondary cases from each genomic cluster, the median pairwise SNP difference between cases in Oxfordshire was 1106 (857–1715). No cluster was within 180 SNPs of another (figure 7).

Within these 13 genomically defined clusters, 11 of 26 transmission events had been previously identified by epidemiological investigation, with none exceeding two SNPs. Nine of 11 transmission events were within a household, including four between family members born in high-incidence countries (G5–E3, G8–E5, G10–E7, and G13–E11) and one between one family member born in a high-incidence country and another in the UK (G10–E7; figure 5). The two non-household cases were linked within a school (G9–E8) and in the community (G11–E10; figure 5). In the retrospective review of the 15 epidemiologically unpredicted links, three were associated with the same homeless shelter (G4), one was related to time spent in the same prison (G4), and two had nearby addresses and shared cultural backgrounds (G3 and G6; appendix). No retrospective explanation could be found for the remaining nine links, including four between patients born in high-incidence and low-incidence countries (figure 5). In all but one case (G6, two patients with smear-negative pulmonary disease), the clusters containing these possible, but epidemiologically unconfirmed transmissions involved at least one patient with smear-positive pulmonary tuberculosis. Of the seven clusters that had household transmissions, five also had genomic links to non-household members not identified on contact tracing (figure 5; appendix).

We noted 17 epidemiologically identified but genomically unconfirmed transmission events. Of these, three were genomically unrelated (22, 721, and 1746 SNPs), 12 could not be assessed because of culture-negative disease, and two because of sample preparation problems (figure 5). The patient who was genomically separated from family members by 22 SNPs migrated to the UK 4 years after the other cases were diagnosed, making direct transmission very unlikely. However, a distance of 22 SNPs is consistent with a dominant circulating clone in the family’s region of origin as a common source.^5^ A similar explanation might apply to two patients separated by 17 SNPs, born in different countries in east Africa but not epidemiologically linked (figure 5).

Using WGS with a 12 SNP threshold as the gold standard, epidemiological investigation had a sensitivity of 0·42 (95% CI 0·23–0·63) and specificity of 0·99 (0·96–1·0) for detection of transmissions. The sensitivity of epidemiological investigation was 0·46 (0·25–0·67) with application of a stricter relatedness threshold of five SNPs (specificity 0·99 [0·96–1·0]), and 0·59 (0·33–0·82) with a one SNP threshold (0·98 [0·96–0·99]).

All Oxfordshire isolates were compared with previously reported sequences from 254 patients within epidemiologically identified clusters in the Midlands.^5^ One Oxfordshire patient with *M bovis* was within two SNPs of the nearest patient in a Midlands *Mycobacterium bovis* outbreak, and one cluster (G2) of four Oxfordshire patients was within nine SNPs of an *M tuberculosis* cluster in the Midlands (clusters 11 and five, respectively in Walker and colleagues^5^). No epidemiological links spanning these geographical boundaries were previously suspected in either case, although patients in the clusters had similar social risk factors.

## Discussion

Over 6 years, 2007–12, we noted 26 (11%) of 246 genomically defined links between evaluable cases. Although more patients with tuberculosis were born in high-incidence than in low-incidence countries, those born in low-incidence countries, predominantly the UK, were more likely to be part of a genomically defined cluster (panel).

Of the 384 cases identified, 269 had microbiologically confirmed disease and of these 247 had isolates that were sequenced. Pairwise SNP distances were used to identify plausible transmissions, using a threshold of 12 SNPs as indication of the maximum pathogen genetic diversity previously noted within hosts and between epidemiologically related hosts.^5^ A bimodal picture emerged with 24 genomic distances spanning zero to four SNPs, 218 longer than 30 SNPs, and only four between five and 30 SNPs, in keeping with previous findings that most patients in a transmission chain are within five SNPs of another patient.^5,19-21^

Although 67% of patients were born in a high-incidence country, these patients were less likely to have pulmonary disease and more likely to be genetically independent of other cases than were patients born in low-incidence countries. This result was also noted in previous studies based on lower resolution fingerprinting methods, although these also suggested an overall rate of transmission twice that identified here.^17,18,22^ Our findings suggest that most patients born in high-incidence countries reactivate latent infection acquired abroad and are not involved in local onward transmission. Unrestricted access to diagnostic and treatment services through the UK National Health Service (NHS) is likely to have contributed to this public health success. As previously noted in other European settings, most migrants to the UK were diagnosed within 5 years of arrival.^23^ The patchwork nature of new-entrant screening in the UK (individual data not available)^24^ stresses the importance of unrestricted access to health care in the early post-migration period in particular. Although health care is freely available to all UK residents, the services seem to have been less effective in controlling disease in patients born in low-incidence countries. Possible explanations might be that the excess of social risk factors in these patients led to inadequate health-care-seeking behaviour, or that health-care professionals investigate other diagnoses before diagnosing tuberculosis in this population. Both out comes could lead to increased periods of infectivity and hence greater onward transmission.

There are several limitations to this study. Like all typing methods, WGS cannot be used to ascertain the source of culture-negative cases. Similarly, we were unable to assess the amount of transmission leading to latent tuberculosis, as data for interferon-γ release assay results could not be linked back to specific contact tracing investigations with confidence. However, 45 (73%) of 62 patients with sputum-smear-positive disease could not be genomically linked to any other case of active disease in Oxfordshire between 2007–12, which supports the intervention programme being fairly successful. MIRU-VNTR typing was only introduced routinely in the UK in 2010, and between 2010–12 MIRU-VNTR cluster investigations were recommended if clusters reached a defined threshold size or contained cases with defined risk factors. In the study population, no additional epidemiological links were identified when cluster investigation was done with this approach. Because the superior resolution of WGS has already been shown,^5-7^ we did not attempt a further comparison. Were the UK-based social networks of recent migrants to span larger geographical distances than were those of long-term residents, then recent migrants might more frequently be linked to transregional rather than regional outbreaks. However, the two genomic links we made to the Midlands involved patients born in low-incidence, not high-incidence, countries.

Our study has several advantages for the future use of WGS. We identified 15 plausible but previously unrecognised transmissions within Oxfordshire. Several of these additional transmissions were from epidemiologically identified household outbreaks to other non-household members. Had these links been identified in near-to-real-time, more intensive investigation might have shown other important routes of transmission, possibly resulting in public health action.^25^ By genomically linking patients in Oxfordshire and the Midlands we also show the potential for identifying previously unrecognised transmission across public health regions. This technique is restricted only by the size of the database for comparison, and not by geographical boundaries, so it could be applied to extend future contact investigations across larger regions.

In this low-incidence setting, the burden of disease was largely sustained by cases infected either outside of the county or the period of study, and onward transmission within the region was associated with birth in a low-incidence rather than a high-incidence setting. Measures targeted at disease control would therefore best be focused on screening new entrants from high-incidence settings for active and latent disease and on improving diagnosis in and access to primary health care for the hard-to-reach groups. In view of these findings, suggested policy interventions aimed at restricting access to NHS care for new entrants, many born in high-incidence countries, raise concerns that disease control could be jeopardised. Effective investigation, diagnosis, and treatment must remain the priorities.

## Supplementary Material

Appendix

## Figures and Tables

**Table:** Associations between country of birth and tuberculosis characteristics and epidemiological or genomic clustering

	Patients with data available	Patients born in low-incidence countries	Patients born in high-incidence countries	Odds ratio* (95% CI)	p value
Pulmonary disease	380 (99%)	78/125 (62%)	119/254 (47%)	1·8 (1·2–2·9)	0·009

Social risk factor	261 (68%)	23/87 (26%)	13/174 (7%)	4·4 (2·0–9·4)	<0·0001

Culture positive disease	377 (98%)	81/125 (65%)	186/252 (74%)	0·6 (0·4–0·99)	0·045

Paediatric disease (age <18 years)	384 (100%)	16/125 (13%)	8/255 (3%)	4·8 (2·0–11·5)	0·001

Epidemiological cluster					
All evaluable patients	384 (100%)	25/125 (20%)	21/255 (8%)	3·3 (1·7–6·3)	<0·0001
Social risk factor data available (not adjusted for social risk factors)	261 (68%)	14/87 (16%)	11/174 (6%)	3·3 (1·4–7·8)	0·006
Social risk factor data available (adjusted for social risk factors)	261 (68%)	14/87 (16%)	11/174 (6%)	3·0 (1·2–7·2)	0·016

Whole-genome-sequencing cluster					
All evaluable patients	247 (64%)	24/74 (32%)	15/172 (9%)	5·8 (2·7–12·4)	<0·0001
Social risk factor data available (not adjusted for social risk factors)	164 (43%)	14/53 (26%)	8/117 (7%)	6·4 (2·2–18·8)	0·001
Social risk factor data available (adjusted for social risk factors)	164 (43%)	14/53 (26%)	8/117 (7%)	4·8 (1·6–14·8)	0·006

Data are number (%) or n/N (%), unless otherwise indicated. Denominators were the numbers of patients for whom data were available for the variables that were being compared.

* For low-incidence countries versus high-incidence countries of birth and calculated with multivariable logistic regression, adjusted for age and sex (just sex for children), and for social risk factors where indicated. Social risk factors are at least one of the following: homelessness, drug or alcohol misuse, or time spent in prison.

## *Panel:* Research in context

### Systematic review

We searched PubMed with the key words “tuberculosis”, and “whole genome sequencing”, “population based”, “transmission”, or “epidemiology” for population-based molecular-epidemiology studies of tuberculosis published in English before October, 2013. Studies of typing with restriction fragment length polymorphism (RFLP) have been reviewed by Houben and Glynn,^16^ whereas Allix-Beguec^17^ and Roetzer^18^ investigated unselected populations by use of the more contemporary typing method mycobacterial interspersed repetitive unit variable number tandem repeat. More recent studies of the genetic diversity of *Mycobacterium tuberculosis* within and between hosts in outbreak settings have shown superior resolution of whole-genome sequencing (WGS) over the previous fingerprinting methods.^5-7,19-21^ However, to our knowledge this is the first population-based study in which the potential of WGS is used to more accurately quantify transmission of tuberculosis within a defined locality over a fixed period.

### Interpretation

In our study, metrics of genetic relatedness derived from recent WGS-based tuberculosis transmission studies were applied to an unselected population for the first time. Because inferences about recent transmission can be made from WGS data with greater certainty than has hitherto been possible using molecular fingerprinting techniques, we can describe the local epidemiology of tuberculosis with unprecedented clarity. This approach has enabled us to assess the effectiveness of current tuberculosis control measures and to provide data to inform the design of future public health interventions. Because patients born in low-incidence countries (mainly the UK) are more likely to be involved in recent transmission chains than are patients born in high-incidence countries, we emphasise the need for systematic new entrant screening and continued free access to health care for all.
