# Supplementary material for: Assessment of Mycobacterium tuberculosis transmission in Oxfordshire, UK, 2007–12, with whole pathogen genome sequences: an observational study
Source: Lancet Respir Med. Author manuscript; Available in PMC 2015 Sep 16. (PMC4571080; doi:10.1016/S2213-2600(14)70027-X)
Supplement: Appendix [file NIHMS64957-supplement-Appendix.pdf]

THE LANCET Respiratory Medicine

Supplementary appendix

This appendix formed part of the original submission and has been peer reviewed. We post it as supplied by the authors.

Supplement to: Walker TM, Lalor MK, Broda A, et al. Assessment of Mycobacterium tuberculosis transmission in Oxfordshire, UK, 2007–12, with whole pathogen genome sequences: an observational study. *Lancet Respir Med* 2014; published online March 4. http://dx.doi.org/10.1016/S2213-2600(14)70027-X.

Supplementary material:

(Assessment of *Mycobacterium tuberculosis* transmission in Oxfordshire, UK, 2007-2012 with whole pathogen genome sequences: an observational study)

Supplementary Table 1: Links between patients are shown by WGS cluster (G1-13, together with SNP distances and results of epidemiological investigations. The number of links in each cluster is equal to the number of patients, minus one. Epidemiological connectedness was defined as shared time and space. The nature of the confirmed (known to have shared time and space) or hypothesised (not known to have shared time and space) epidemiological connection is listed.

WGS Cluster	Reconstructed patient links	SNPs	Epidemiologically linked (known to have shared time and space)	Epidemiological connection (either confirmed or hypothesised)
G 1	P347 - P136	7	No	
G 2	P44 - P33	2	No	
G 2	P33 - P119	3	No	
G 2	P119 - P249	7	No	
G 3	P406 - P460	0	No	Same country of birth / neighbourhood
G 4	P139 - P343	1	Yes	Household / family
G 4	P428 - P446	1	No	Night shelter
G 4	P376 - P428	4	No	Night shelter
G 4	P428 - P445	2	No	Night shelter
G 4	P428 - P139	3	No	
G 4	P376 - P410	0	Yes	Household / family
G 5	P386 - P387	0	Yes	Household / family
G 5	P387 - P64	0	No	
G 5	P167 - P386	1	No	
G 6	P49 - P104	3	No	Same country of birth / neighbourhood
G 7	P330 - P479	1	No	Prison
G 7	P330 - P331	2	Yes	Household / family
G 8	P486 - P96	0	Yes	Household / family
G 8	P96 - P175	0	No	
G 9	P84 - P184	0	Yes	Household / family
G 9	P84 - P27	1	Yes	School
G 10	P22 - P202	0	Yes	Household / family
G 10	P202 - P372	0	Yes	Household / family
G 11	P165 - P469	0	Yes	Social connection
G 12	P164 - P374	0	No	
G 13	P52 - P43	1	Yes	Household / family

Supplementary Table 2: Associations between UK birth and other low incidence countries of birth, and disease characteristics and epidemiological or genomic clustering

Outcome	N (%) with data available	Outcome in patients born in the UK	Outcome in patients born in other low-incidence countries	Odds ratio (UK-born vs other low-incidence country of birth)	95% CI	p-value
Pulmonary disease	125 (100%)	63/103 (61%)	15/22 (68%)	0.8	0·3-2·2	0·66
Social risk factor	87 (70%)	19/70 (27%)	4/17 (24%)	1·5	0·4-5·2	0·55
Culture positive disease	125 (100%)	64/103 (62%)	17/22 (77%)	0·5	0·2-1·6	0·26
Paediatric disease (age<18)	125 (100%)	16/103 (16%)	0/22 (0%)			
Epidemiological cluster	125 (100%)	22/103 (21%)	3/22 (14%)	1·2	0·3-5·0	0·76
Epidemiological cluster if data on social risk available (not adjusted for social risk)	87 (70%)	11/70 (16%)	3/17 (18%)	0·6	0·1-2·8	0·54
Adjusted for social risk	87 (70%)	11/70 (16%)	3/17 (18%)	0·55	0·1-2·6	0·45
WGS cluster	74 (59%)	22/58 (38%)	2/16 (12%)	6·6	1·3-34·4	0·033
WGS cluster if data on social risk available (not adjusted for social risk)	52 (42%)	12/39 (31%)	2/13 (15%)	2·9	0·5-16·0	0·22
Adjusted for social risk	52 (42%)	12/39 (31%)	2/13 (15%)	2·0	0·3-12·4	0·45

Note: odds ratios based on multi-variable logistic regression, adjusted for age and gender, and also for social risk factors where indicated.

Supplementary figure 1: Age distribution of cases according to incidence in country of birth

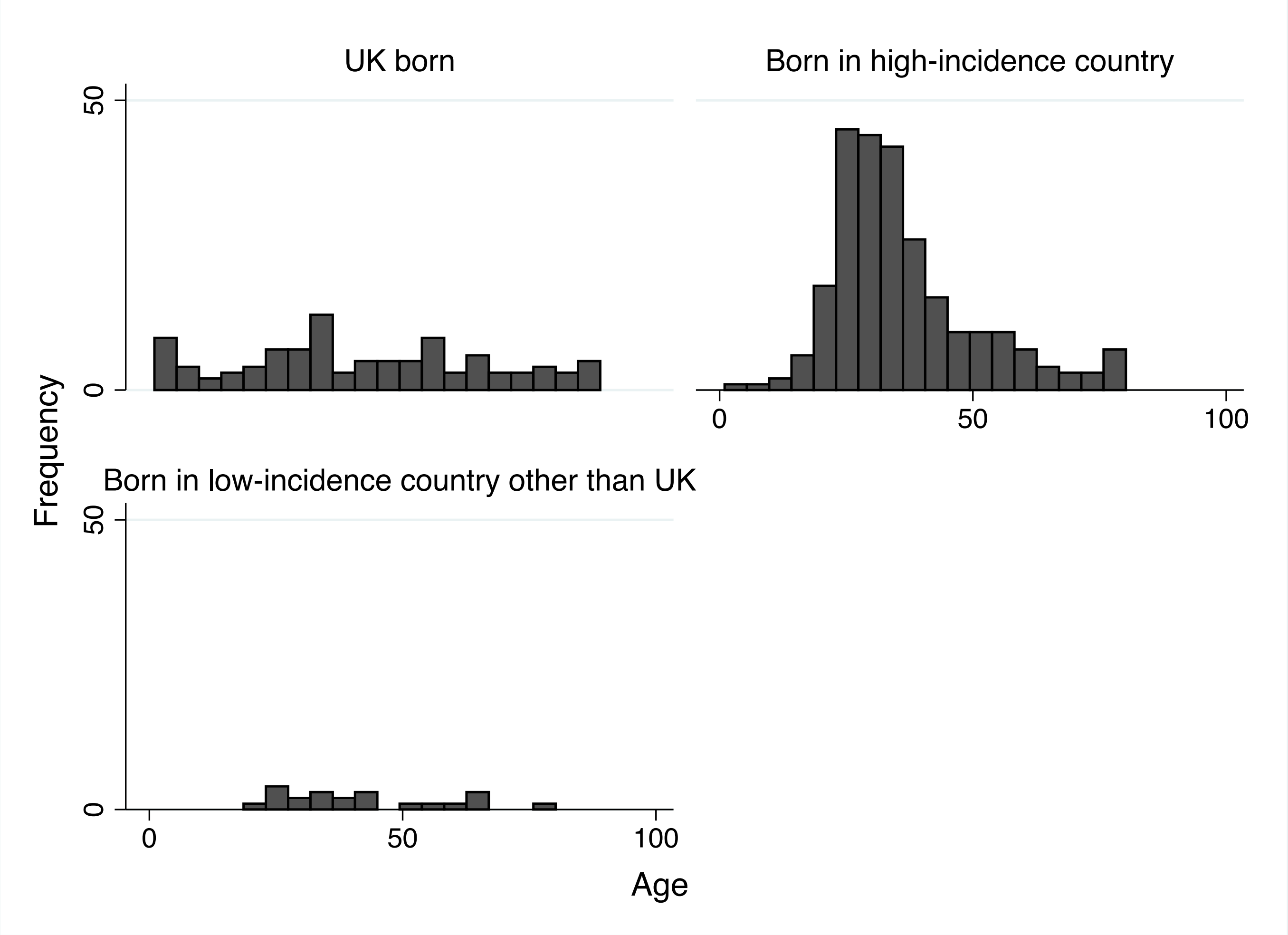

Appendix

All cases and sequenced isolates included in the study

Patient	ENA accession number	Year of diagnosis	Postcode	Incidence in country of birth	Epidemiological cluster	WGS cluster
OxTbPat_1	ERS389395	2009	OX4	High	.	.
OxTbPat_2		2011	OX4	High	.	.
OxTbPat_3	ERS389348	2009	OX3	Low	.	.
OxTbPat_4	ERS389512	2012	OX1	High	.	.
OxTbPat_5	ERS389496	2008	OX4	High	.	.
OxTbPat_6	ERS389555	2011	OX4	Low	.	.
OxTbPat_7		2008	OX4	High	.	.
OxTbPat_8		2012	OX3	Low	.	.
OxTbPat_9	ERS389588	2010	OX3	Low	.	.

Appendix

OxTbPat_10		2009	OX5	High	.	.
OxTbPat_12		2009	OX4	High	.	.
OxTbPat_13	ERS389568	2008	OX4	High	.	.
OxTbPat_14		2011	OX3	High	.	.
OxTbPat_15		2007	OX4	Low	11	.
OxTbPat_16		2007	OX3	Low	.	.
OxTbPat_20	ERS389386	2011	OX16	High	.	.
OxTbPat_22	ERS389485	2007	OX16	Low	7	10
OxTbPat_23	ERS389400	2008	OX17	High	.	.
OxTbPat_24	ERS389441	2008	OX14	High	.	.
OxTbPat_26		2009	OX5	High	.	.
OxTbPat_27	ERS389580	2011	OX16	Low	8	9

Appendix

OxTbPat_29		2011	SN6	.	.	.
OxTbPat_30		2010	OX4	High	.	.
OxTbPat_31	ERS389449	2010	OX3	Low	.	.
OxTbPat_32		2010	RG4	Low	.	.
OxTbPat_33	ERS389465	2011	OX3	Low	.	2
OxTbPat_35	ERS389459	2009	OX3	High	.	.
OxTbPat_36	ERS389520	2010	OX4	High	.	.
OxTbPat_37	ERS389475	2008	OX12	Low	.	.
OxTbPat_38		2008	OX16	High	.	.
OxTbPat_39		2008	OX2	High	.	.
OxTbPat_41	ERS389366	2011	OX4	High	.	.
OxTbPat_43	ERS389476	2007	OX4	High	11	13

OxTbPat_44	ERS389397	2007	OX3	Low	.	2
OxTbPat_45		2009	OX3	High	.	.
OxTbPat_46		2010	OX2	High	.	.
OxTbPat_47	ERS389558	2012	OX4	High	.	.
OxTbPat_48	ERS389484	2007	OX4	High	.	.
OxTbPat_49	ERS389464	2007	OX4	High	.	6
OxTbPat_50	ERS389572	2009	OX4	Low	.	.
OxTbPat_51	ERS389567	2008	RG9	High	.	.
OxTbPat_52	ERS389378	2007	OX3	High	11	13
OxTbPat_53	ERS389356	2009	OX4	High	.	.
OxTbPat_54		2011	OX3	High	.	.
OxTbPat_55	ERS389442	2008	OX29	Low	.	.

Appendix

OxTbPat_56	ERS389583	2012	OX4	High	.	.
OxTbPat_57	ERS389396	2011	OX16	High	.	.
OxTbPat_58	ERS389428	2007	OX16	High	14	.
OxTbPat_59		2007	OX16	High	7	.
OxTbPat_60		2007	OX29	Low	.	.
OxTbPat_62		2012	OX11	Low	.	.
OxTbPat_63		2009	OX15	Low	.	.
OxTbPat_64	ERS389472	2012	OX1	Low	.	5
OxTbPat_65		2010	OX18	Low	.	.
OxTbPat_66	ERS389385	2011	OX49	Low	.	.
OxTbPat_67		2009	OX10	Low	.	.
OxTbPat_68	ERS389463	2009	OX4	High	.	.

OxTbPat_69	ERS389402	2007	OX7	Low	.	.
OxTbPat_72		2009	OX4	High	.	.
OxTbPat_73	ERS389541	2012	OX11	High	.	.
OxTbPat_74		2011	OX4	High	.	.
OxTbPat_76	ERS389560	2012	OX4	High	.	.
OxTbPat_77		2010	OX4	High	.	.
OxTbPat_78		2009	OX4	High	.	.
OxTbPat_79		2009	SN6	High	.	.
OxTbPat_80		2011	OX20	Low	.	.
OxTbPat_81	ERS389519	2011	OX25	Low	.	.
OxTbPat_82	ERS389566	2008	RG9	Low	.	.
OxTbPat_83	ERS389438	2007	OX16	High	.	.

OxTbPat_84	ERS389517	2011	OX16	Low	8	9
OxTbPat_85		2008	OX4	High	.	.
OxTbPat_86		2008	OX4	High	.	.
OxTbPat_87	ERS389479	2008	OX4	High	.	.
OxTbPat_88		2011	OX4	High	.	.
OxTbPat_89		2012	OX12	Low	.	.
OxTbPat_91	ERS389564	2007	OX3	High	.	.
OxTbPat_92	ERS389372	2008	OX13	High	.	.
OxTbPat_93		2011	OX2	Low	.	.
OxTbPat_94		2009	OX4	High	.	.
OxTbPat_95		2007	OX14	Low	.	.
OxTbPat_96	ERS389480	2008	OX5	High	5	8

Appendix

OxTbPat_97	ERS389403	2010	OX3	High	18	.
OxTbPat_98	ERS389514	2012	OX4	High	.	.
OxTbPat_100		2009	OX4	High	.	.
OxTbPat_101		2010	OX4	High	.	.
OxTbPat_102	ERS389436	2009	OX4	Low	.	.
OxTbPat_103	ERS389542	2012	OX2	High	.	.
OxTbPat_104	ERS389408	2008	OX4	High	.	6
OxTbPat_105		2011	OX4	High	.	.
OxTbPat_108		2012	MK18	Low	.	.
OxTbPat_109	ERS389527	2009	OX33	High	.	.
OxTbPat_110		2011	OX28	Low	.	.
OxTbPat_111	ERS389437	2007	OX15	Low	.	.

OxTbPat_112	ERS389462	2009	OX4	High	.	.
OxTbPat_113	ERS389488	2009	OX4	High	.	.
OxTbPat_114	ERS389563	2007	OX4	High	.	.
OxTbPat_117	ERS389455	2007	OX4	High	.	.
OxTbPat_118	ERS389507	2012	OX3	High	.	.
OxTbPat_119	ERS389516	2010	OX4	High	.	2
OxTbPat_121		2012	OX4	High	.	.
OxTbPat_122	ERS389543	2012	OX26	Low	.	.
OxTbPat_123	ERS389536	2012	OX4	Low	.	.
OxTbPat_124	ERS389511	2012	OX10	Low	.	.
OxTbPat_125		2008	OX15	High	.	.
OxTbPat_126		2010	OX3	Low	.	.

OxTbPat_129	ERS389373	2010	OX4	High	.	.
OxTbPat_130	ERS389365	2010	OX4	High	.	.
OxTbPat_133	ERS389467	2011	OX4	High	.	.
OxTbPat_134		2007	OX9	Low	.	.
OxTbPat_136	ERS389535	2011	OX27	Low	.	1
OxTbPat_137	ERS389582	2012	RG8	Low	.	.
OxTbPat_138		2012	OX26	Low	2	.
OxTbPat_139	ERS389554	2012	OX26	Low	2	4
OxTbPat_140		2011	OX12	High	.	.
OxTbPat_142	ERS389358	2009	OX3	Low	.	.
OxTbPat_143		2009	OX3	High	15	.
OxTbPat_144		2009	OX3	High	15	.

OxTbPat_145	ERS389506	2012	OX2	Low	.	.
OxTbPat_146		2012	OX14	High	.	.
OxTbPat_147	ERS389447	2010	OX4	High	.	.
OxTbPat_148		2008	RG9	High	.	.
OxTbPat_149	ERS389569	2008	OX4	High	.	.
OxTbPat_150		2007	OX4	High	.	.
OxTbPat_151	ERS389359	2009	OX4	High	.	.
OxTbPat_152	ERS389571	2009	RG9	High	.	.
OxTbPat_155	ERS389468	2011	OX29	High	.	.
OxTbPat_156	ERS389361	2010	OX4	High	.	.
OxTbPat_157		2008	OX16	High	.	.
OxTbPat_158		2009	OX14	Low	.	.

Appendix

OxTbPat_159	ERS389578	2010	OX10	Low	.	.
OxTbPat_160	ERS389487	2007	OX4	High	.	.
OxTbPat_161		2011	OX5	High	.	.
OxTbPat_162		2009	OX4	High	.	.
OxTbPat_163	ERS389391	2007	OX3	High	.	.
OxTbPat_164	ERS389491	2007	OX16	High	.	12
OxTbPat_165	ERS389575	2011	OX5	Low	10	11
OxTbPat_166		2007	RG9	Low	.	.
OxTbPat_167	ERS389446	2007	OX16	High	.	5
OxTbPat_168		2009	OX4	Low	.	.
OxTbPat_169	ERS389371	2010	OX16	High	.	.
OxTbPat_170	ERS389544	2012	OX4	Low	.	.

OxTbPat_171		2007	OX16	Low	.	.
OxTbPat_172	ERS389389	2007	OX4	High	.	.
OxTbPat_173		2007	OX1	High	.	.
OxTbPat_175	ERS389353	2009	OX4	Low	4	8
OxTbPat_177		2008	OX16	High	.	.
OxTbPat_178	ERS389381	2009	OX16	High	.	.
OxTbPat_179		2009	OX4	High	.	.
OxTbPat_180		2007	OX1	High	.	.
OxTbPat_181	ERS389383	2009	OX4	High	.	.
OxTbPat_182		2007	OX25	Low	.	.
OxTbPat_183		2009	OX3	High	.	.
OxTbPat_184	ERS389533	2011	OX16	Low	8	9

OxTbPat_186	ERS389562	2007	OX44	High	.	.
OxTbPat_187	ERS389344	2011	OX13	Low	.	.
OxTbPat_188	ERS389433	2011	OX14	High	.	.
OxTbPat_189	ERS389574	2011	OX1	Low	.	.
OxTbPat_190	ERS389374	2010	OX16	High	.	.
OxTbPat_191		2009	OX10	High	.	.
OxTbPat_192	ERS389382	2007	OX2	High	.	.
OxTbPat_193		2008	OX4	High	.	.
OxTbPat_194	ERS389431	2007	OX4	High	.	.
OxTbPat_195		2007	OX4	High	.	.
OxTbPat_197		2012	OX3	High	.	.
OxTbPat_199	ERS389590	2011	OX26	Low	13	.

OxTbPat_200	ERS389503	2012	OX3	High	.	.
OxTbPat_201		2007	OX16	High	7	.
OxTbPat_202	ERS389529	2007	OX16	High	7	10
OxTbPat_203		2007	OX16	High	7	.
OxTbPat_204		2012	OX16	High	12	.
OxTbPat_205		2011	OX4	Low	.	.
OxTbPat_206		2010	OX3	High	.	.
OxTbPat_207	ERS389577	2011	OX9	High	.	.
OxTbPat_208		2012	OX16	Low	.	.
OxTbPat_209		2007	OX3	Low	.	.
OxTbPat_211	ERS389493	2007	OX12	High	.	.
OxTbPat_212		2011	OX3	High	.	.

OxTbPat_213	ERS389432	2007	OX29	High	.	.
OxTbPat_214	ERS389581	2012	OX2	High	.	.
OxTbPat_215	ERS389367	2010	OX1	High	.	.
OxTbPat_216		2007	OX4	High	.	.
OxTbPat_218		2011	OX4	High	.	.
OxTbPat_219	ERS389490	2010	OX4	High	.	.
OxTbPat_220	ERS389591	2010	OX16	High	.	.
OxTbPat_221	ERS389424	2011	OX4	High	.	.
OxTbPat_222	ERS389534	2011	OX16	High	.	.
OxTbPat_223	ERS389392	2007	OX4	High	.	.
OxTbPat_224	ERS389407	2008	OX26	Low	13	.
OxTbPat_226	ERS389483	2010	OX16	High	.	.

OxTbPat_227	ERS389363	2008	OX26	High	.	.
OxTbPat_229	ERS389474	2007	OX4	High	.	.
OxTbPat_230	ERS389427	2009	OX3	High	.	.
OxTbPat_231	ERS389525	2008	OX2	High	.	.
OxTbPat_232	ERS389401	2007	OX26	High	.	.
OxTbPat_234	ERS389345	2010	OX4	High	.	.
OxTbPat_237	ERS389482	2011	OX3	High	.	.
OxTbPat_238	ERS389553	2012	OX4	High	.	.
OxTbPat_239	ERS389552	2012	OX4	High	.	.
OxTbPat_241	ERS389362	2010	OX3	High	.	.
OxTbPat_242	ERS389492	2007	OX16	Low	.	.
OxTbPat_243	ERS389570	2008	OX10	High	.	.

OxTbPat_245		2012	OX3	High	.	.
OxTbPat_246		2009	OX12	Low	.	.
OxTbPat_247	ERS389498	2012	OX14	High	.	.
OxTbPat_248	ERS389510	2011	OX2	High	.	.
OxTbPat_249	ERS389515	2012	OX3	Low	.	2
OxTbPat_250	ERS389579	2012	OX1	Low	.	.
OxTbPat_251	ERS389350	2011	OX4	High	.	.
OxTbPat_252	ERS389419	2010	OX2	Low	.	.
OxTbPat_253		2008	OX3	High	.	.
OxTbPat_254	ERS389393	2010	OX16	Low	.	.
OxTbPat_255	ERS389349	2009	OX3	High	.	.
OxTbPat_257		2010	OX12	Low	.	.

OxTbPat_259	ERS389509	2012	OX7	Low	16	.
OxTbPat_260	ERS389379	2007	OX3	Low	.	.
OxTbPat_263	ERS389409	2011	OX4	High	.	.
OxTbPat_264	ERS389380	2009	OX1	High	.	.
OxTbPat_267	ERS389387	2010	OX2	High	.	.
OxTbPat_268		2010	OX4	High	.	.
OxTbPat_269	ERS389346	2011	OX17	Low	.	.
OxTbPat_270	ERS389354	2007	OX5	High	.	.
OxTbPat_272		2007	OX2	.	.	.
OxTbPat_273	ERS389347	2010	OX12	Low	.	.
OxTbPat_274		2012	OX18	High	.	.
OxTbPat_275		2008	OX4	High	.	.

OxTbPat_277		2009	SN7	High	.	.
OxTbPat_279		2012	OX3	Low	.	.
OxTbPat_281		2007	OX3	High	.	.
OxTbPat_282	ERS389499	2012	OX16	High	.	.
OxTbPat_284	ERS389369	2008	OX29	High	.	.
OxTbPat_285	ERS389481	2010	OX26	High	.	.
OxTbPat_288	ERS389410	2011	OX3	High	.	.
OxTbPat_289	ERS389414	2007	OX16	Low	.	.
OxTbPat_290	ERS389357	2008	OX16	High	.	.
OxTbPat_291	ERS389351	2011	OX4	High	.	.
OxTbPat_292	ERS389444	2008	OX3	High	.	.
OxTbPat_299	ERS389497	2008	OX3	High	.	.

OxTbPat_301		2012	OX3	High	.	.
OxTbPat_304		2008	OX12	High	.	.
OxTbPat_305	ERS389423	2008	OX3	High	.	.
OxTbPat_306	ERS389384	2009	OX14	Low	.	.
OxTbPat_307	ERS389445	2008	OX16	High	.	.
OxTbPat_308	ERS389470	2011	OX3	High	.	.
OxTbPat_309	ERS389411	2011	OX3	High	.	.
OxTbPat_311		2007	OX4	High	.	.
OxTbPat_312	ERS389398	2007	OX4	High	.	.
OxTbPat_313	ERS389375	2010	OX3	High	.	.
OxTbPat_314		2009	RG9	Low	.	.
OxTbPat_315	ERS389450	2011	OX12	High	.	.

OxTbPat_316	ERS389415	2010	SN7	Low	.	.
OxTbPat_317	ERS389500	2012	OX4	Low	.	.
OxTbPat_318	ERS389390	2007	OX4	High	.	.
OxTbPat_319	ERS389523	2010	OX15	Low	.	.
OxTbPat_320	ERS389589	2010	OX4	High	.	.
OxTbPat_322		2007	OX33	High	.	.
OxTbPat_323	ERS389557	2012	OX29	Low	.	.
OxTbPat_325	ERS389430	2009	OX1	High	.	.
OxTbPat_326	ERS389451	2010	OX3	High	.	.
OxTbPat_327		2012	OX4	High	.	.
OxTbPat_328	ERS389421	2007	OX2	High	.	.
OxTbPat_330	ERS389434	2007	OX16	Low	6	7

OxTbPat_331	ERS389341	2007	OX16	Low	6	7
OxTbPat_333	ERS389478	2007	OX4	High	.	.
OxTbPat_334	ERS389477	2008	OX7	Low	.	.
OxTbPat_335		2011	OX4	Low	.	.
OxTbPat_336	ERS389539	2012	OX26	High	.	.
OxTbPat_337	ERS389584	2008	OX16	High	.	.
OxTbPat_339		2010	OX14	High	.	.
OxTbPat_341	ERS389550	2012	OX4	High	.	.
OxTbPat_342	ERS389549	2012	OX4	High	.	.
OxTbPat_343	ERS389545	2012	OX26	Low	2	4
OxTbPat_347	ERS389489	2007	OX14	Low	.	1
OxTbPat_348	ERS389439	2008	OX4	High	.	.

OxTbPat_349	ERS389518	2010	OX7	High	.	.
OxTbPat_351	ERS389404	2009	OX2	High	.	.
OxTbPat_352		2010	OX5	Low	.	.
OxTbPat_354	ERS389548	2012	OX4	High	.	.
OxTbPat_355	ERS389452	2011	OX1	High	.	.
OxTbPat_356	ERS389454	2010	OX10	High	.	.
OxTbPat_357	ERS389538	2012	OX3	High	.	.
OxTbPat_358	ERS389530	2010	OX4	High	.	.
OxTbPat_359	ERS389587	2008	OX33	Low	.	.
OxTbPat_360		2007	OX14	Low	.	.
OxTbPat_361	ERS389559	2012	ZZ	High	.	.
OxTbPat_362	ERS389406	2008	OX4	High	.	.

OxTbPat_365		2011	RG4	Low	.	.
OxTbPat_366	ERS389461	2009	OX26	Low	.	.
OxTbPat_367	ERS389412	2011	OX26	High	.	.
OxTbPat_368	ERS389340	2011	OX26	High	.	.
OxTbPat_369	ERS389592	2012	OX16	High	.	.
OxTbPat_371		2007	OX16	Low	7	.
OxTbPat_372	ERS389494	2008	OX16	High	7	10
OxTbPat_373	ERS389376	2010	OX16	High	9	.
OxTbPat_374	ERS389504	2012	OX16	Low	9	12
OxTbPat_375	ERS389526	2007	OX4	Low	.	.
OxTbPat_376	ERS389343	2011	OX4	Low	1	4
OxTbPat_377	ERS389522	2011	OX3	High	11	.

OxTbPat_378		2010	OX4	High	.	.
OxTbPat_380	ERS389426	2010	OX16	High	.	.
OxTbPat_381		2008	OX3	High	.	.
OxTbPat_382	ERS389443	2008	OX3	High	.	.
OxTbPat_384	ERS389405	2008	OX4	High	.	.
OxTbPat_385	ERS389399	2007	OX4	High	.	.
OxTbPat_386	ERS389469	2011	OX4	High	3	5
OxTbPat_387	ERS389471	2011	OX4	High	3	5
OxTbPat_391		2010	OX26	High	.	.
OxTbPat_392	ERS389377	2009	OX16	Low	.	.
OxTbPat_394	ERS389360	2009	OX4	High	.	.
OxTbPat_395	ERS389429	2007	OX2	High	.	.

OxTbPat_396		2008	OX4	High	.	.
OxTbPat_398	ERS389355	2009	OX25	High	.	.
OxTbPat_399	ERS389422	2010	OX2	High	.	.
OxTbPat_400		2010	OX18	Low	.	.
OxTbPat_401	ERS389460	2008	OX2	High	.	.
OxTbPat_402	ERS389440	2008	OX4	High	.	.
OxTbPat_403	ERS389513	2012	OX26	High	.	.
OxTbPat_404	ERS389495	2011	OX16	High	.	.
OxTbPat_405	ERS389416	2007	OX4	High	.	.
OxTbPat_406	ERS389524	2010	OX3	High	.	3
OxTbPat_407		2010	OX4	High	.	.
OxTbPat_408		2010	OX14	High	.	.

OxTbPat_410	ERS389342	2011	OX4	Low	1	4
OxTbPat_412		2011	OX4	High	.	.
OxTbPat_414	ERS389576	2011	SN6	.	.	.
OxTbPat_415	ERS389352	2011	OX3	Low	.	.
OxTbPat_417	ERS389413	2009	OX11	High	.	.
OxTbPat_418	ERS389458	2007	OX4	High	.	.
OxTbPat_419		2011	OX25	Low	.	.
OxTbPat_420	ERS389501	2012	GL56	Low	.	.
OxTbPat_422	ERS389435	2007	OX4	High	.	.
OxTbPat_423	ERS389425	2011	OX3	High	.	.
OxTbPat_425		2007	OX4	.	.	.
OxTbPat_426	ERS389417	2011	OX4	High	.	.

OxTbPat_427	ERS389505	2012	OX16	Low	.	.
OxTbPat_428	ERS389448	2008	OX4	Low	.	4
OxTbPat_429	ERS389586	2010	OX5	High	.	.
OxTbPat_430		2011	OX2	High	.	.
OxTbPat_431		2009	OX4	High	.	.
OxTbPat_432		2011	OX3	High	.	.
OxTbPat_434		2009	OX11	Low	.	.
OxTbPat_436	ERS389573	2009	RG4	High	.	.
OxTbPat_438		2012	OX16	Low	.	.
OxTbPat_439	ERS389546	2012	OX4	Low	.	.
OxTbPat_440		2011	RG8	Low	.	.
OxTbPat_442	ERS389453	2009	OX10	High	.	.

OxTbPat_443	ERS389540	2012	OX3	High	.	.
OxTbPat_444		2010	OX2	High	.	.
OxTbPat_445	ERS389521	2008	OX3	Low	.	4
OxTbPat_446	ERS389502	2012	OX4	Low	.	4
OxTbPat_449		2007	OX2	High	.	.
OxTbPat_450	ERS389388	2007	OX4	High	.	.
OxTbPat_451		2007	OX2	High	.	.
OxTbPat_452	ERS389532	2011	OX3	High	.	.
OxTbPat_453		2012	OX4	High	.	.
OxTbPat_454	ERS389508	2012	OX4	High	.	.
OxTbPat_455	ERS389418	2007	OX26	Low	.	.
OxTbPat_456	ERS389551	2012	OX1	High	.	.

OxTbPat_457		2009	OX3	High	.	.
OxTbPat_458	ERS389370	2008	OX3	High	.	.
OxTbPat_459	ERS389528	2009	OX4	High	.	.
OxTbPat_460	ERS389368	2008	OX3	High	.	3
OxTbPat_462	ERS389457	2010	OX4	High	.	.
OxTbPat_466		2012	OX3	High	.	.
OxTbPat_467	ERS389565	2007	OX4	High	.	.
OxTbPat_468		2011	OX3	Low	.	.
OxTbPat_469	ERS389420	2007	OX5	Low	10	11
OxTbPat_471		2012	OX16	High	12	.
OxTbPat_473		2009	OX28	Low	.	.
OxTbPat_475	ERS389473	2011	OX2	Low	.	.

OxTbPat_477		2009	OX3	Low	.	.
OxTbPat_479	ERS389486	2011	OX25	Low	.	7
OxTbPat_482		2009	OX16	Low	.	.
OxTbPat_483	ERS389394	2009	OX2	High	.	.
OxTbPat_484		2008	OX16	Low	14	.
OxTbPat_485	ERS389364	2011	OX3	High	.	.
OxTbPat_486	ERS389456	2009	OX5	High	5	8
OxTbPat_487		2008	OX13	Low	.	.
OxTbPat_488		2008	OX2	Low	.	.
OxTbPat_489		2012	OX4	Low	17	.
OxTbPat_490	ERS389466	2012	OX4	High	17	.
OxTbPat_491		2011	OX3	High	18	.

OxTbPat_529	2012	OX4	Low	4	.
OxTbPat_530	2012	GL56	Low	16	.
OxTbPat_531	2012	GL56	Low	16	.

Appendix
